# Supplementary figures and images for: Enhancer prediction in the human genome by probabilistic modelling of the chromatin feature patterns
Source: BMC Bioinformatics. 2020 Jul 20;21:317. doi: 10.1186/s12859-020-03621-3 (PMC7370432; doi:10.1186/s12859-020-03621-3)

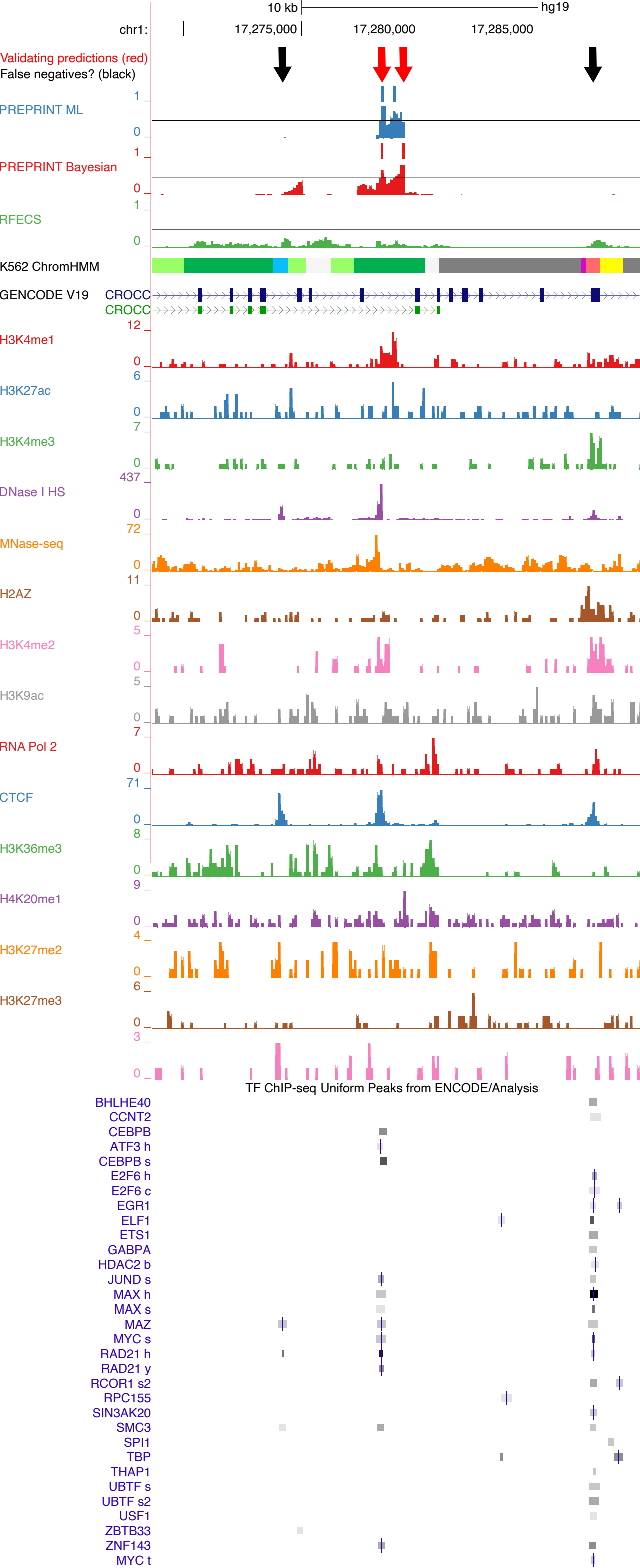

Supplement: Supplementary file 3 — Additional file 3 The full genome browser example figure of the K562 cell line data. PDF of size 199 kB. [file 12859_2020_3621_MOESM3_ESM.pdf]

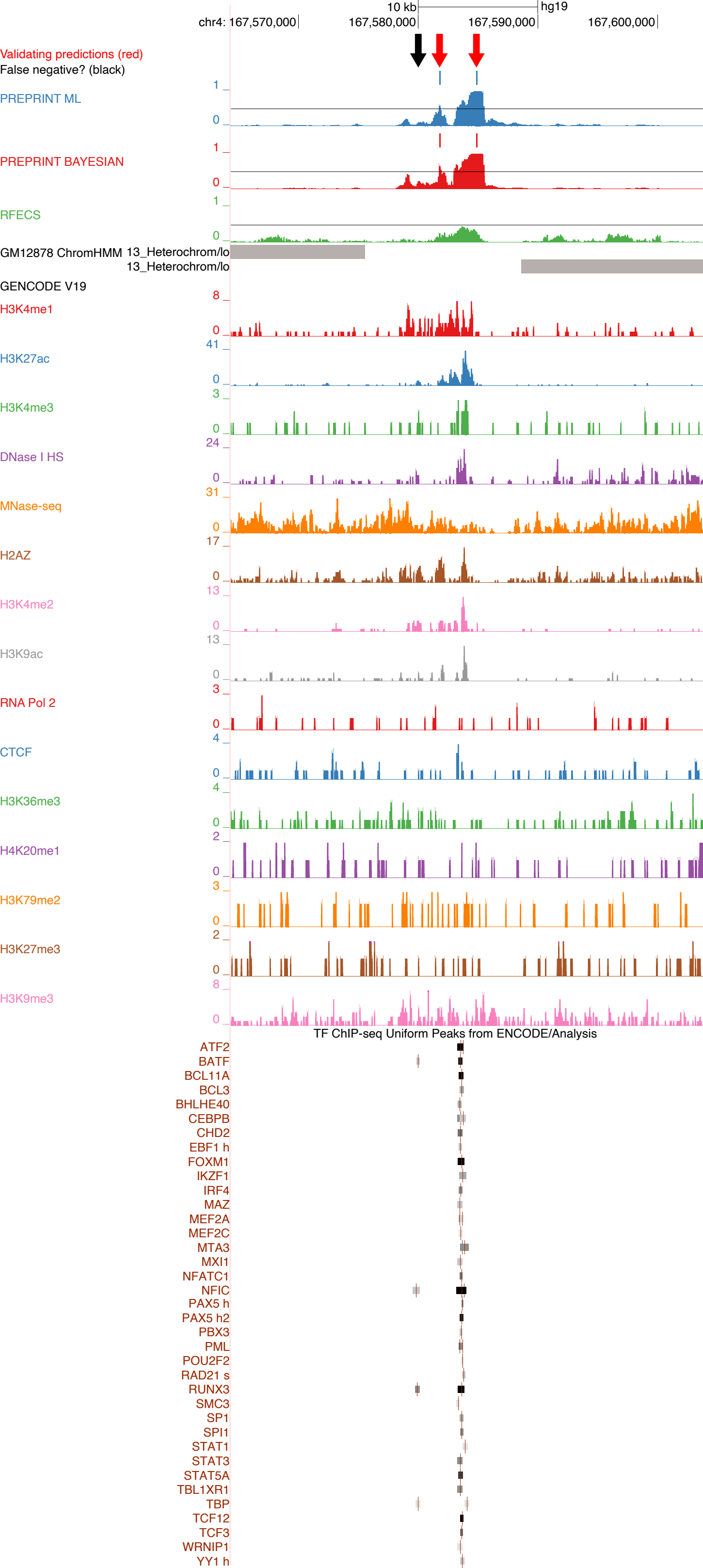

Supplement: Supplementary file 4 — Additional file 4 The full genome browser example figure of the GM12878 cell line data. PDF of size 217 kB. [file 12859_2020_3621_MOESM4_ESM.pdf]
